# Traumatic Brain Injury Alters Methionine Metabolism: Implications for Pathophysiology

**DOI:** 10.3389/fnsys.2016.00036

**Published:** 2016-04-29

**Authors:** Pramod K. Dash, Georgene W. Hergenroeder, Cameron B. Jeter, H. Alex Choi, Nobuhide Kobori, Anthony N. Moore

**Affiliations:** ^1^Department of Neurobiology and Anatomy, UTHealth McGovern Medical SchoolHouston, TX, USA; ^2^The Vivian L. Smith Department of Neurosurgery, UTHealth McGovern Medical SchoolHouston, TX, USA; ^3^Department of Diagnostic and Biomedical Sciences, University of Texas School of DentistryHouston, TX, USA

**Keywords:** concussion, epigenetic changes, metabolomics, protein methylation, S-adenosylmethionine, transsulfuration

## Abstract

Methionine is an essential proteinogenic amino acid that is obtained from the diet. In addition to its requirement for protein biosynthesis, methionine is metabolized to generate metabolites that play key roles in a number of cellular functions. Metabolism of methionine via the transmethylation pathway generates S-adenosylmethionine (SAM) that serves as the principal methyl (−CH_3_) donor for DNA and histone methyltransferases (MTs) to regulate epigenetic changes in gene expression. SAM is also required for methylation of other cellular proteins that serve various functions and phosphatidylcholine synthesis that participate in cellular signaling. Under conditions of oxidative stress, homocysteine (which is derived from SAM) enters the transsulfuration pathway to generate glutathione, an important cytoprotective molecule against oxidative damage. As both experimental and clinical studies have shown that traumatic brain injury (TBI) alters DNA and histone methylation and causes oxidative stress, we examined if TBI alters the plasma levels of methionine and its metabolites in human patients. Blood samples were collected from healthy volunteers (HV; *n* = 20) and patients with mild TBI (mTBI; GCS > 12; *n* = 20) or severe TBI (sTBI; GCS < 8; *n* = 20) within the first 24 h of injury. The levels of methionine and its metabolites in the plasma samples were analyzed by either liquid chromatography-mass spectrometry or gas chromatography-mass spectrometry (LC-MS or GC-MS). sTBI decreased the levels of methionine, SAM, betaine and 2-methylglycine as compared to HV, indicating a decrease in metabolism through the transmethylation cycle. In addition, precursors for the generation of glutathione, cysteine and glycine were also found to be decreased as were intermediate metabolites of the gamma-glutamyl cycle (gamma-glutamyl amino acids and 5-oxoproline). mTBI also decreased the levels of methionine, α-ketobutyrate, 2 hydroxybutyrate and glycine, albeit to lesser degrees than detected in the sTBI group. Taken together, these results suggest that decreased levels of methionine and its metabolic products are likely to alter cellular function in multiple organs at a systems level.

## Introduction

It has been appreciated for more than 30 years that the resting metabolic expenditure of the severely injured brain is almost 40% higher than that of the non-injured brain, and is associated with a negative nitrogen balance (the difference between nitrogen uptake and nitrogen excretion), suggesting increased protein catabolism (Clifton et al., 1985). Increasing enteral nutrition to compensate for the enhanced nitrogen excretion has shown only partial success (Clifton et al., 1985), possibly due to poor gut absorption and/or impaired gut motility. In particular, insufficient availability of essential amino acids (primarily obtained from the diet) can not only negatively influence cell survival and function in the injured brain, but also can impair the function of other organs as well. In addition to serving as the building blocks for proteins, amino acids and their metabolites play critical roles in various cellular and physiological functions (e.g., regulation of cerebral perfusion by the arginine metabolite nitric oxide). Although a few studies have measured amino acid levels in traumatic brain injury (TBI) patients (Aquilani et al., 2000; Jeter et al., 2012, 2013; Vuille-Dit-Bille et al., 2012), whether the metabolic products of these amino acids are altered in TBI patients is largely unknown.

**Figure 1:**
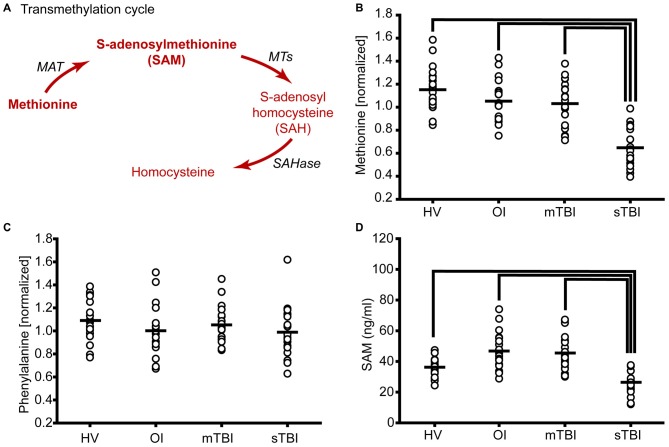
**Severe traumatic brain injury (sTBI) reduces plasma methionine and metabolism through the transmethylation pathway. (A)** Schematic showing the metabolism of methionine via the transmethylation pathway. Molecules detected and measured are presented in **bold** text. Key enzymes are indicated by *italic* text. The plasma levels of **(B)** methionine were significantly reduced as a result of TBI. **(C)** S-adenosylmethionine (SAM) was significantly reduced in sTBI compared to healthy volunteers (HV) and patients with a mild TBI (mTBI). When the data were segregated by gender, similar changes in both **(D)** methionine and **(E)** SAM were observed in males and females. MAT: methionine adenosyltransferase; MTs: methyltransferases; SAHase: S-adenosyl-L-homocysteine hydrolase. Horizontal bar indicates mean. *p < 0.05.

Methionine is an essential amino acid for protein synthesis and is often incorporated as the first amino acid. Metabolism of methionine occurs by two primary pathways: the transmethylation and the transsulfuration (Figures 1A, 3A, 4A). The transmethylation pathway generates S-adenosylmethionine (SAM), an important methyl donor for the methylation of lipids, proteins, and nucleotides. For example, SAM-dependent DNA and histone methyltransferases (MTs) have been found to be key enzymes in the epigenetic regulation of gene expression (Cantoni, 1975; Bird, 2007). SAM also donates methyl groups for the synthesis of phosphatidylcholine, a major phospholipid component of cell membranes and an important signaling molecule for both intra- and inter-cellular communication (Hirata and Axelrod, 1980). In addition to generating SAM, methionine is required for the synthesis of glutathione via the transsulfuration pathway. Under conditions of stress, cells use glutathione to scavenge reactive oxygen species (ROS) in order to reduce oxidative damage. Both experimental and clinical studies have shown that TBI causes oxidative damage to the injured brain, which may be related to decreases in glutathione availability (Povlishock and Kontos, 1992; Bayir et al., 2002; Singh et al., 2006; Bains and Hall, 2012). Thus, decreases in the levels of methionine and/or its metabolic products may underlie oxidative damage and the progression of TBI pathology and outcome.

**Figure 2:**
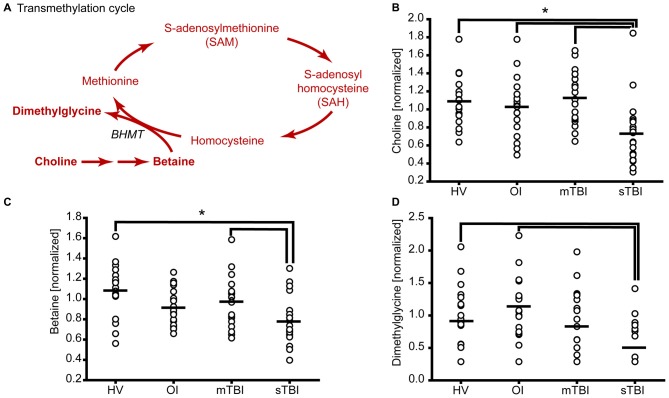
**sTBI reduces the levels of methyl donors for methionine remethylation. (A)** Schematic showing the methylation of homocysteine to generate methionine. Molecules detected and measured are presented in **bold** text. Key enzymes are indicated by *italic* text. The plasma levels of the methyl donors **(B)** choline and **(C)** betaine used in methionine remethylation were significantly reduced in sTBI. **(D)** The side-product of methionine regeneration, dimethylglycine, is significantly reduced as a result of sTBI. BHMT: betaine-homocysteine S-methyltransferase. Horizontal bar indicates mean. *p < 0.05.

**Figure 3:**
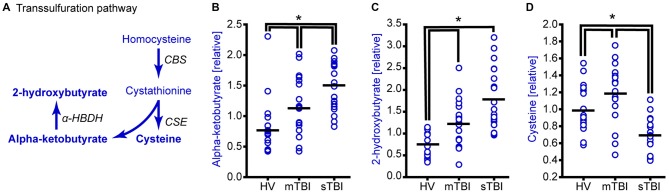
**TBI alters methionine metabolism through the transsulfuration pathway. (A)** Drawing of the transsulfuration pathway in which homocysteine is converted to cysteine and alpha-ketobutyrate. Alpha-ketobutyrate is reduced to generate 2-hydroxybutyrate. Molecules detected and measured in the current study are presented in **bold** text. Key enzymes are indicated by *italic* text. Although the plasma levels of both **(B)** alpha-ketobutyrate and **(C)** 2-hydroxybutyrate were significantly elevated in the plasma of sTBI patients compared to other groups, the plasma levels of **(D)** cysteine were significantly decreased in sTBI patients. In contrast, the levels of cysteine were significantly increased in the mTBI group. HV: healthy volunteers; mTBI: mild TBI patients; CBS: cystathionine-β-synthase; CSE: cystathionine γ-lyase; α-HBDH: alpha-hydroxybutyrate dehydrogenase. Horizontal bar indicates mean. *p < 0.05.

**Figure 4:**
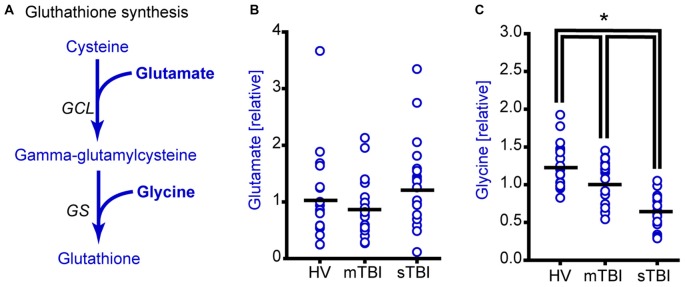
**Plasma glycine, but not glutamate levels are reduced as a result of sTBI. (A)** Simplified drawing showing the production of glutathione from cysteine, glycine and glutamate. Glutamate cysteine ligase (GCL) catalyzes the reaction between glutamate and cysteine to produce gamma-glutamylcysteine. Glutathione synthase (GS) converts gamma-glutamylcysteine and glycine to generate glutathione. Molecules detected and measured in the current study are presented in **bold** text. Key enzymes are indicated by *italic* text. **(B)** Glutamate levels did not change across any of the subject groups. Horizontal bar indicates mean. **(C)** Plasma glycine levels in individual subjects from HV mTBI, and sTBI are shown. The levels of glycine are significantly reduced in the plasma of sTBI patients compared to all other groups. *p < 0.05.

In the present study, we measured the levels of methionine and several of its metabolites in plasma samples collected within the first 24 h of their injury from patients who experienced either a severe (GCS ≤ 8) or mild (GCS > 12) TBI (sTBI or mTBI). Plasma samples from healthy volunteers (HV) were used as controls. An acute time point for sample collection was chosen as experimental studies have shown that robust oxidative damage and cell death occurs during this period. Our results indicate that both mild and severe TBI cause significant reductions in plasma methionine levels. A decrease in the transmethylation product SAM was observed in sTBI patients, as were the plasma levels of choline, betaine, and dimethylglycine. The levels of the transsulfuration metabolite cysteine, as well as the gamma-glutamyl cycle metabolites (gamma-glutamyl amino acids and 5-oxoproline), were also found to be reduced after sTBI. Taken together, these results demonstrate that TBI decreases methionine and its key metabolites, which may alter the function of multiple organs, and suggest that supplementation of methionine metabolites may be beneficial for sTBI patients.

## Materials and Methods

### Recruitment of Study Subjects

The University of Texas Health Science Center at Houston Committee for the Protection of Human Subjects approved the human subject protocol in accordance with the Declaration of Helsinki. In total, 60 subjects were recruited and provided written informed consented for participation in this study. All subjects were between 14–57 years of age and provided consent or proxy consent for their participation in this study. None of the subjects had drug dependency, or had active infections. Twenty sTBI (GCS ≤ 8), 20 mTBI (GCS ≥ 12), and 20 HV were recruited for this study. mTBI had no abnormalities on head computed tomography (CT) scans, but experienced one or more of the following: loss of consciousness, post-traumatic amnesia, altered mental status, neurologic deficits, or seizure. Demographic and clinical information on the study subjects is provided in Table 1. The predominant causes of all injuries were motor vehicle accidents and falls.

**Table 1:** **Demographic and clinical data for study subjects**.

Group	Healthy volunteers	Mild TBI	Severe TBI
Number of subjects	20	20	20
GCS (24 h of injury)	NA	14.85 ± 0.37	3.65 ± 1.2 (intubated)
Injury Severity Score	NA	5.4 ± 2.4	27.5 ± 8.2
Age (years)	25.2 ± 6.7	36.1 ± 13.3	25.8 ± 9.8
Female/Male	4/16	6/14	4/16
Hispanic ethnicity	6	3	4
Race
White	16	18	16
African American	3	2	3
Asian	1	0	1

*TBI, traumatic brain injury; values in mean ± standard deviation*.

### Sample Collection and Analysis

Seventeen of the 20 HV did not eat after midnight, the night before sample collection (at least 12 h before sample collection). The mTBI subjects had a last recorded meal that was 7.5 ± 4.4 h prior to sample collection. sTBI samples were collected 15.07 ± 5.0 h after the time of their injury. Blood samples were obtained within the first 24 h after injury and were coded to protect anonymity. Samples were collected in potassium EDTA tubes (Becton Dickinson, Franklin Lakes, NJ, USA), placed on ice, and processed within an hour of draw. Plasma was isolated by centrifugation at 4°C as described by the vendor. Aliquots were prepared and frozen at −80°C until needed. Plasma was processed by Metabolon, Inc. (Durham, NC, USA) using a proprietary series of extractions designed to increase the sensitivity of small molecule detection. Samples were placed briefly on a TurboVap^®^ (Caliper Technologies Corp., Hopkinton, MA, USA) to remove any organic solvent. Each sample was then frozen and dried under vacuum. The samples were analyzed by liquid chromatography-mass spectrometry (LC-MS) or gas chromatography-mass spectrometry (GC-MS) depending on the analyte being interrogated. Methionine and its metabolites were identified by comparison to purified standards. A selection of quality control compounds was added to every sample. Relative levels of each metabolite were quantified using Metabolon’s proprietary peak integration software. The integrated peak values from all subjects were averaged and used for normalizing the values for each individual subject.

### Enzyme-Linked Immunosorbent Assays (ELISAs)

SAM was measured using a competitive ELISA as described by the vendor (BioVendor, Asheville, NC, USA). A standard curve for calculating the abundance of SAM was generated by serial dilution of a purified standard. The range of the standards was based on the vendors’ instructions. Standards and plasma samples (50 μl) were added to a 96-well plate containing immobilized antibodies specific to SAM. Biotinylated SAM was immediately added to the well, after which the plate was incubated at 37°C for 1 h. After extensive washing, a streptavidin-horseradish peroxidase conjugate was added and incubated for 30 min. The plate was washed, and developed using tetramethylbenzidine (TMB). The reaction was terminated by the addition of 2N sulfuric acid. The optical density was measured using a microplate reader at 450 nm. Concentration of SAM in the plasma was calculated using a 4-parameter logistic curve.

### Statistical Analysis

Data was initially evaluated using a Shapiro-Wilk normality test, followed by a one-way analysis of variance (ANOVA) across the four subject groups. Any data found to not have a normal distribution was analyzed using a Kruskal-Wallis ANOVA on ranks. Groups with differences were identified using a Dunn’s pairwise comparison as the *post hoc* test. Differences were considered significant at *p* < 0.05, with groups with altered levels identified using critical *p*-values calculated after compensation for multiple comparisons.

## Results

Methionine is metabolized by two primary metabolic cycles: the transmethylation pathway to generate SAM and homocysteine (Figure 1A); and the transsulfuration cycle to generate glutathione (Figures 3A, 4A). While not all individual metabolites of these cycles could be detected and quantified, intermediate metabolites for all three cycles were detected. Based on the changes in the levels of these intermediate metabolites, alterations in the flux of these three pathways can be inferred.

### The Methionine (Transmethylation) Cycle

The transmethylation cycle involves the metabolism of methionine to generate SAM, S-adenosylhomocysteine (SAH), and homocysteine (Figure 1A). When the relative plasma levels of methionine were measured in patients with either sTBI (GCS < 8) or mTBI (GCS > 12) and compared to HV, a significant change across the groups was detected (*F* = 44.01, *p* < 0.001). *Post hoc* analysis revealed a significant reduction of plasma methionine in both mild and severe TBI patients relative to HV, with greater reductions detected in the sTBI group (Figure 1B). In contrast, the relative levels of phenylalanine, a second essential amino acid, were found to be unchanged across all three groups (*F* = 1.82, *p* = 0.17). Methionine is converted to SAM by L-methionine S-adenosyltransferase (MAT). Although SAM was not detected by LC/GC-MS, we used a competitive ELISA to measure the levels of this important methyl donor. Figure 1C shows concentration of SAM in individual samples. When analyzed by a one-way ANOVA on Ranks, a significant decrease (*H* = 29.44, *p* < 0.001) in plasma SAM levels was detected in the sTBI patients compared to the other groups. As it has been reported that there are gender differences in amino acid metabolism (Lamont et al., 2003), we next parceled the methionine and SAM results to examine sex-related differences. As TBI occurs predominately in males (4:1 male to female ratio), our recruited study subjects followed this distribution resulting in 4–6 females in each of our study groups (Table 1). However, even with this small number of females, we observed that the changes in methionine appear to be independent of gender with both males and females showing significant decreases (*F* = 17.58, *p* < 0.001) in plasma methionine in the sTBI group (Figure 1D). However, due to the reduction in number of subjects in each group, the difference between the mTBI group and HV was no longer statistically significant. Plasma SAM (*F* = 9.70, *p* < 0.001) was also found to change similarly in males and females (Figure 1E), although more error-prone in the females due to the small number of study subjects. No significant differences between males and females within any group were detected.

A number of methyltransferases use SAM as a methyl donor for DNA, protein and lipid methylation and convert SAM to SAH (Cantoni, 1975; Grillo and Colombatto, 2008). SAH is then converted by SAH hydrolase to homocysteine, which can be used as a substrate to regenerate methionine (Figure 2A). In this pathway, choline is metabolized to betaine, which then acts as the methyl donor for the methylation of homocysteine by betaine homocysteine methyltransferase (BHMT). During this reaction, homocysteine is converted to methionine and dimethylglycine. Figure 2B shows the relative levels of choline in the plasma samples of individual subjects from all three groups. Plasma choline levels in the sTBI group were found to be significantly reduced as compared to the other groups (*H* = 18.64; *p* < 0.001). Betaine (trimethylglycine) is synthesized from choline via a multistep enzymatic pathway. The plasma levels of betaine were found to be significantly reduced (*F* = 8.42; *p* < 0.001) in the sTBI patients relative to the HV and mTBI group (Figure 2C). When the plasma levels of dimethylglycine were interrogated, a significant decrease (*H* = 8.76 *p* = 0.013) in its plasma levels were detected in the samples from sTBI patients compared to HV, but not the mTBI group (Figure 2D). Taken together, the decreased levels of these metabolites indicated decreased flux through the transmethylation pathway.

### The Transsulfuration Pathway

Transsulfuration of homocysteine is essentially an irreversible reaction that generates cystathionine (via cystathionine β-synthase), which is further metabolized by cystathionine gamma-ligase to produce cysteine and alpha-ketobutyrate. Alpha-ketobutyrate is then reduced by α-hydroxybutyrate dehydrogenase to generate 2-hydroxybutyrate (Figure 3A). Although the relative levels of both α-ketobutyrate (Figure 3B; *H* = 22.05, *p* < 0.001) and 2-hydroxybutyrate (Figure 3C; *H* = 28.65, *p* < 0.001) were found to be significantly increased in the plasma of sTBI patients, cysteine levels showed a significant reduction (Figure 3D; *F* = 20.23, *p* < 0.001). Interestingly, the levels of cysteine in the plasma of mTBI was found to be significantly increased relative to HV.

Glutamate cysteine ligase (GCL) ligates cysteine with glutamate to generate gamma-glutamylcysteine that is then combined with glycine by glutathione synthase (GS) to generate glutathione (Figure 4A). Although the relative plasma levels of glutamate did not significantly change across groups (Figure 4B; *H* = 2.21, *p* = 0.331), the plasma levels of glycine were found to be significantly reduced in both the mild and severe TBI patients (Figure 4C; *F* = 27.61, *p* < 0.001).

### The Gamma-Glutamyl Cycle

**Figure 5:**
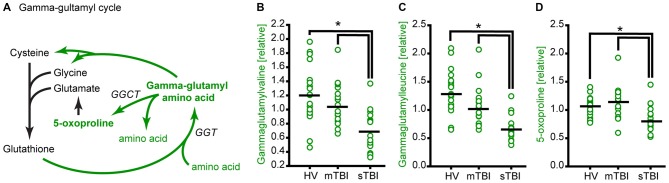
**TBI reduces glutathione recycling through the gamma-glutamyl cycle. (A)** Simplified schematic showing the enzymes and intermediates for the gamma-glutamyl cycle. Molecules detected and measured in the current study are presented in **bold** text. Key enzymes are indicated by *italic* text. The levels of the gamma-glutamyl amino acids **(B)** gamma-glutamylvaline and **(C)** gamma-glutamylleucine, and the intermediate **(D)** 5-oxoproline are significantly reduced in the plasma of sTBI patients compared to HV, and mTBI groups. GGT: gamma-glutamyl transpeptidase; GGCT: gamma-glutamyl cyclotransferase. Horizontal bar indicates mean. *p < 0.05.

A decrease in cysteine and glycine would be expected to result in a decrease in the levels of glutathione. Unfortunately, plasma glutathione could not be detected by our mass spectrometry analysis. In addition to its role in the reduction of oxidative stress, glutathione is used to facilitate the transport of amino acids into cells via the gamma-glutamyl cycle (Orlowski and Meister, 1970). This occurs via the conversion of amino acids to gamma-glutamyl amino acids by gamma-glutamyl transpeptidase (GGT). The release of amino acids from gamma-glutamyl amino acids by gamma-glutamyl cyclotransferase (GGCT) generates 5-oxoproline as an intermediate (Figure 5A). Thus, a decrease in glutathione availability would be anticipated to result in a decrease in gamma-glutamyl amino acids and 5-oxoproline. The relative levels of gamma-glutamylvaline (Figure 5B; *F* = 13.54, *p* < 0.001), gamma-glutamylleucine (Figure 5C; *H* = 30.48, *p* < 0.001), gamma-glutamylisoleucine (*H* = 23.60, *p* < 0.001), gamma-glutamyltyrosine (*H* = 14.12, *p* < 0.001), and gamma-glutamylphenylalanine (*F* = 4.56, *p* = 0.015) were found to be significantly decreased in the plasma of sTBI patients. Consistent with the decrease in gamma-glutamyl amino acids, 5-oxoproline was found to be significantly reduced in the plasma of sTBI patients compared to other groups (Figure 5D; *H* = 17.66, *p* < 0.001).

## Discussion

**Figure 6:**
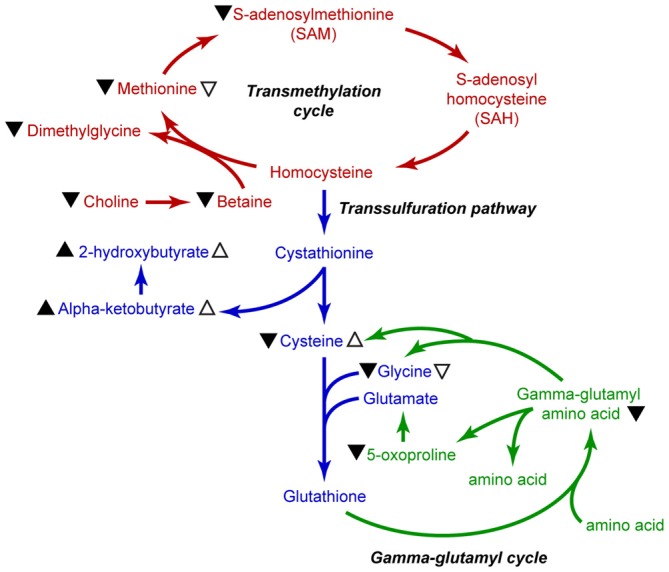
**Summary illustration of methionine metabolic pathways.** Metabolites of the transmethylation pathway are indicated in red, the transsulfuration pathway is indicated in blue, and the gamma-glutamyl cycle is shown in green. Black arrowhead (▴) indicates changes in metabolite levels in the plasma of sTBI patients. Open arrows (▵) indicate significant changes measured in the mTBI patients as compared to HV. Up arrowheads indicate increased levels, whereas down arrowheads indicate decreased levels.

The essential amino acid methionine not only acts as a building block for protein synthesis, but also serves as the substrate for the synthesis of key molecules such as SAM and glutathione. As such, changes in the levels of methionine metabolites could impact a number of biological processes such as epigenetic regulation of gene expression and cytoprotection. Our measurements of methionine and its metabolites in plasma samples from sTBI, mTBI, and HV revealed four key findings: (1) the relative plasma levels of methionine and SAM are significantly reduced in sTBI patients; (2) the levels of cysteine and glycine, the precursors for the synthesis of glutathione, are also reduced in sTBI patients; (3) in contrast to that observed in sTBI patients, the plasma levels of cysteine were significantly elevated in mTBI; and (4) the relative levels of several gamma-glutamyl amino acids and 5-oxoproline are significantly reduced in the plasma of sTBI patients. These findings are summarized in Figure 6. Taken together, our findings suggest that the reduced availability of methionine, SAM and glutathione as a result of sTBI may alter multiple cellular processes including protein synthesis, epigenetic regulation of gene expression, cytoprotection, and the cellular transport of amino acids in multiple organs including the injured brain.

As methionine is an essential amino acid, its decrease in both mild and severe TBI patients could have resulted from a reduction in dietary intake. However, the time from last meal was comparable for the HV and sTBI groups, with all groups having not eaten within 7 h of sample collection. Thus, the changes in methionine levels we observed in TBI patients may not be solely due to lack of food intake. Previous clinical studies have shown that sTBI causes a nitrogen imbalance in which nitrogen excretion exceeds nitrogen uptake (Clifton et al., 1985), suggesting increased protein break-down. Attempts to restore nitrogen balance using enteral feeding found that replacement of 17 g N/day yielded a nitrogen balance of −9.2 g/day, whereas replacement of 29 g N/day restored nitrogen balance to −5 g/day (Clifton et al., 1985). Given that the human body requires up to 1.5 g protein/kg/day in order to maintain lean body mass and normal protein synthesis (Bistrian and Babineau, 1998), higher protein loads are likely required to preserve muscle mass. However, although enteral hyperalimentation can partially restore metabolic expenditure in sTBI patients, feeding rarely achieves nitrogen balance resulting in weight loss of up to 15% by the second week after injury (Clifton et al., 1985). As the principal source of excreted nitrogen is urea, and urea is generated from the deamination of amino acids, this suggests that circulating amino acids levels should also be reduced in TBI patients. Consistent with this, it has been reported that sTBI patients have low levels of serum amino acids, including essential amino acids such as branched-chain amino acids (BCAAs), histidine, and methionine when measured 2 months after injury (Aquilani et al., 2000). In addition to chronic reductions in amino acid levels, the reduced levels of some amino acids have been seen as early as 24 h after TBI (Jeter et al., 2012, 2013). Irrespective of the mechanism by which methionine levels are reduced, decreased methionine availability would be expected to reduce its brain levels. In support of this, it has been demonstrated that the administration of intravenous amino acids results in an increase in their levels in the brain (Lajtha and Mela, 1961).

Only a few clinical studies have examined the consequences of amino acid supplementation after TBI. Ott et al. (1988) compared the effects of two different intravenous amino acid formulations on amino acid levels and nitrogen balance in sTBI patients. Their results indicated that positive nitrogen balance could be achieved with early administration of some combinations of amino acids, although neither the specific amino acids responsible for this improvement, nor their effect on outcome was assessed (Ott et al., 1988). BCAAs play important roles in regulating protein synthesis, gluconeogenesis, and energy metabolism as well as functioning as a major source of nitrogen for producing glutamine and nitric oxide (Fernstrom, 2005; Jeter et al., 2013). Experimental studies have shown that dietary supplementation of BCAAs improves cognitive function and can reduce injury-associated sleep disturbances (Lim et al., 2013; Elkind et al., 2015). Aquilani et al. (2000) tested if supplementation of BCAAs for 15 days during rehabilitation (average time from injury: 2 months) improved outcome, and found that patients receiving BCAAs had improved cognitive function as indicated by a reduction in their Disability Rating Scale (DRS) scores. Nägeli et al. (2014) tested if inclusion of an alanine-glutamine dipeptide as a part of normal feeding (initiated 3 days after injury) could be used to increase plasma and brain glutamine levels without corresponding increases in cerebral glutamate. Although alanine and glutamine levels in the brain were found to be increased in the absence of a net increase in cerebral glutamate, the benefit of this treatment on outcome was not evaluated (Nägeli et al., 2014). At present, it has not been determined if supplementation of methionine would improve outcome in sTBI patients. However, in an animal model of TBI, administration of methionine was found to increase neuronal apoptosis via yet unidentified mechanisms (Akkaya et al., 2014). As we observed that methionine and a number of its metabolites were reduced as a result of TBI, supplementation of these or other intermediates may be beneficial and may reduce complications associated with methionine administration.

SAM is a key methyl group donor for methylation of intracellular molecules including DNA, proteins and phospholipids (Grillo and Colombatto, 2008). A methyl group is transferred to these substrates by different methyl transferases. Methylation of these molecules regulates a number of important biological processes including epigenetic gene regulation (via DNA and/or histone methylation), axonal transport (via microtubule methylation) and plasma membrane integrity and signal (via phospholipid methylation; Cantoni, 1975; Hirata and Axelrod, 1980; Bhaumik et al., 2007; Bird, 2007). Second only to ATP, SAM is one of the most commonly used molecules in living organisms. In experimental models of TBI, changes in substrate methylation have been observed for histones and cellular DNA, and have been suggested to contribute to ongoing pathologies including reactive gliosis and impaired axonal regeneration, as well as contribute to the development of chronic neurodegeneration (Gao et al., 2006; Zhang et al., 2007; Portela and Esteller, 2010; Lardenoije et al., 2015). Several studies have evaluated the consequences of SAM supplementation to treat diseases such as depression, Alzheimer’s disease, tauopathy, liver disease, and various cancers (Purohit et al., 2007; Lu et al., 2009; Papakostas, 2009; Lee et al., 2012; Montgomery et al., 2014). For example, Papakostas (2009) tested if SAM could be used as an adjunct therapy for depressed patients who do not respond to serotonin reuptake inhibitors (SSRIs). By comparison to patients maintained only on SSRIs, those whose received SAM supplementation had higher Hamilton Depression Rating Scale (HAM-D) response and remission rates (Papakostas, 2009), indicating a reduction in depression severity and an increase in wellness, respectively. As our measurements indicate a significant reduction in the relative levels of SAM in the plasma of sTBI patients, SAM supplementation may be useful to treat some of the behavioral problems seen in these patients.

As the brain has a high metabolic activity and high lipid content, it is particularly vulnerable to oxidative damage. Glutathione is the major antioxidant molecule, and a decrease in its levels can exacerbate brain damage. Although intracellular glutathione levels are relatively high, circulating levels of glutathione are low, due to its extremely short half-life (ranging from seconds to minutes; Wendel and Cikryt, 1980). This likely contributed to our inability to detect glutathione in our plasma samples. However, our findings that glutathione precursors cysteine and glycine were significantly reduced, as well as corresponding reductions in gamma-glutamyl amino acids, suggest a decrease in its levels. It is therefore anticipated that supplementation of precursors of glutathione may offer protection. Consistent with this, systemic administration of N-acetylcysteine, in combination with minocycline, improves cognitive function in brain injured animals (Haber et al., 2013).

Amino acids are thought to be transported into the cell via their conversion to gamma-glutamyl amino acids, a process dependent on glutathione (Orlowski and Meister, 1970). Gamma-glutamyl amino acids are then transported into the cytosol where they are processed by gamma-glutamylcyclotransferase to yield free amino acids and 5-oxoproline. As we observed decreased levels of several gamma-glutamyl amino acids, as well as 5-oxoproline, these findings suggest that intracellular transport of amino acids via the gamma-glutamyl cycle is decreased. These decreases, in conjunction with (or resulting from) decreased amino acid levels, could further reduce the intracellular availability of methionine and other amino acids.

In addition to changes in methionine metabolism in sTBI patients, we found a modest, but significant, decrease in plasma methionine levels in mTBI as compared to HV (Figure 1). This suggests that a decrease in methionine following TBI may be related to injury severity. In addition to methionine, we also observed TBI severity related changes in the levels of alpha-ketobutyrate, 2-hydroxybutyrate (Figure 3) and glycine (Figure 4). Interestingly, cysteine levels were found to increase in the plasma of mTBI (Figure 3), whereas its levels decreased in sTBI patients as compared to HV. The reason for this divergence is not clear at present.

A number of weakness need to be considered when interpreting our results. As methionine and its metabolites were measured in the plasma, the source of these molecules cannot be ascertained. Additional weakness of the present study include that the activity and levels of the enzymes for methionine metabolism were not measured. Alterations in their activity, in conjunction with decreased methionine levels, could have given rise to the changes we observed. If supplementation of protein/methionine restores the levels of SAM and other metabolites, this would suggest that the metabolic enzymes of methionine metabolism are not altered. However, this is yet to be examined. Another weakness of our study is that the consequence of SAM reduction on the methylation of cellular proteins, DNA and phospholipids has not been determined. A study by Yi et al. (2000) has reported that an increase in plasma SAM levels is associated with hypomethylation of DNA in lymphocytes. This suggests that the reduced plasma SAM levels we measured in sTBI patients may result in the hypermethylation of DNA and subsequent suppression of gene expression. Finally, although we examined the influence of gender on methionine and SAM levels, the number of females in our study population was small. Thus, a larger group of females would need to be assessed to truly evaluate if there are gender differences in methionine metabolism after TBI.

In summary, our results show that sTBI patients have low levels of circulating methionine, and that methionine metabolites generated via the transmethylation and transsulfuration cycle are significantly reduced. In addition, flux through the gamma-glutamyl cycle is decreased. As methionine and its metabolites are critical for a number of cellular functions and cytoprotection, decreases in their plasma levels may contribute to brain injury pathology. Alternatively, these biochemical changes may serve a protective function in the acute stage of injury, and their persistence may be detrimental. The systemic changes in methionine and metabolite levels we observed may not only influence the function and pathology of the injured brain, but may alter the function of other organs as well.

## Author Contributions

PKD, GWH, CBJ, HAC, NK and ANM all contributed to either the conception, design, acquisition, analysis, or interpretation of data for the manuscript. Further, PKD, GWH, CBJ, HAC, NK and ANM all contributed to either drafting the manuscript or revising it for intellectual content. PKD, GWH, CBJ, HAC, NK and ANM have all approved the version of the manuscript to be published and agree to be accountable for all aspects of the work in ensuring that questions of the accuracy or integrity of any part of the work are appropriately investigated and resolved.

## Funding

This study was supported by Grants from NIH (NS087149), Mission Connect/TIRR Foundation, and the Gilson-Longenbaugh Foundation.

## Conflict of Interest Statement

The authors declare that the research was conducted in the absence of any commercial or financial relationships that could be construed as a potential conflict of interest. The reviewer IN and handling Editor declared their shared affiliation, and the handling Editor states that the process nevertheless met the standards of a fair and objective review.

## Abbreviations

ANOVA: analysis of variance
BCAAs: branched-chain amino acids
BHMT: betaine homocysteine methyltransferase
CT: computed tomography
DRS: Disability Rating Scale
GC-MS: gas chromatography-mass spectrometry
GCL: glutamate cysteine ligase
GCS: Glasgow coma scale
GGT: gamma-glutamyl transpeptidase
GGCT: gamma-glutamyl cyclotransferase
HAM-D: Hamilton depression rating scale
HV: healthy volunteers
LC-MS: liquid chromatography-mass spectrometry
MAT: L-methionine S-adenosyltransferase
mTBI: mild TBI
ROS: reactive oxygen species
SAH: S-adenosylhomocysteine
SAM: S-adenosylmethionine
SSRIs: serotonin reuptake inhibitors
TBI: traumatic brain injury
TMB: tetramethylbenzidine.
